# Assessment of methods for amino acid matrix selection and their use on empirical data shows that ad hoc assumptions for choice of matrix are not justified

**DOI:** 10.1186/1471-2148-6-29

**Published:** 2006-03-24

**Authors:** Thomas M Keane, Christopher J Creevey, Melissa M Pentony, Thomas J Naughton, James O Mclnerney

**Affiliations:** 1 Bioinformatics Laboratory, Department of Biology, National University of Ireland, Maynooth, Co. Kildare, Ireland; 2 Bork Group, EMBL Heidelberg, Heidelberg, Germany; 3 Department of Computer Science, University College London, Gower Street, London, UK; 4 Department of Computer Science, National University of Ireland, Maynooth, Co. Kildare, Ireland

## Abstract

**Background:**

In recent years, model based approaches such as maximum likelihood have become the methods of choice for constructing phylogenies. A number of authors have shown the importance of using adequate substitution models in order to produce accurate phylogenies. In the past, many empirical models of amino acid substitution have been derived using a variety of different methods and protein datasets. These matrices are normally used as surrogates, rather than deriving the maximum likelihood model from the dataset being examined. With few exceptions, selection between alternative matrices has been carried out in an ad hoc manner.

**Results:**

We start by highlighting the potential dangers of arbitrarily choosing protein models by demonstrating an empirical example where a single alignment can produce two topologically different and strongly supported phylogenies using two different arbitrarily-chosen amino acid substitution models. We demonstrate that in simple simulations, statistical methods of model selection are indeed robust and likely to be useful for protein model selection. We have investigated patterns of amino acid substitution among homologous sequences from the three Domains of life and our results show that no single amino acid matrix is optimal for any of the datasets. Perhaps most interestingly, we demonstrate that for two large datasets derived from the proteobacteria and archaea, one of the most favored models in both datasets is a model that was originally derived from retroviral Pol proteins.

**Conclusion:**

This demonstrates that choosing protein models based on their source or method of construction may not be appropriate.

## Background

For a number of years phylogenetic construction has been considered to be a problem of statistical inference. One of the most popular methods of inferring phylogenetic relationships is maximum likelihood (ML). It has often been considered that one of the advantages of ML over parsimony based methods is that it allows for the use of different models of evolution depending on the dataset being examined. Therefore knowing the process of evolution and being able to construct realistic models of evolution is the foundation for being able to infer accurate phylogenetic relationships among species. Currently one of the major challenges in phylogenetics is to accurately model the process of nucleotide or amino acid substitution and to choose among our set of models in order to infer accurate phylogenies. Felsenstein [1] was the first to show in simulations that overly simplistic models that underestimate multiple substitutions can result in inconsistency during phylogeny estimation in certain situations (referred to as the 'Felsenstein zone'). Further simulation studies have shown that even when using ML analysis, underestimation of nucleotide substitutions (as assumed by simpler models) leads to long-branch attraction and inconsistency in the Felsenstein zone [2,3]. These results have also been duplicated in real datasets where the use of inadequate models can lead to long-branch attraction [4,5]. However it was also shown in simulations that violations of the model can also favour the true tree in certain situations (often referred to as the 'Farris zone' or 'inverse-Felsenstein zone') [6,7]. It was later shown in simulations and real data that this can only happen in a very limited number of cases and in general using overly simplistic models should be avoided [3,7,8]. One fact that is most certainly true is that the accurate estimation of node support is strongly affected by the use of simplistic models in simulated and real datsets [9,10]. It is clear that unless we can be totally sure that a dataset fits into one of the categories mentioned above, then the use of overly simplistic (or incorrect) substitution models can negatively bias our phylogenies.

Almost all models of amino acid replacement assume that all amino acids sites evolve independently according to the same Markov process. It is assumed that the Markov process is stationary and homogeneous, so that all rates of substitution are constant across time. Each of the protein substitution models consists of a 20 × 20 instantaneous rate matrix which includes the set of original amino acid frequencies (*π*_*i*_) obtained from the dataset that was used to generate the model. The (*π*_*i*_) values represent the equilibrium or stationary frequencies of the 20 amino acids and the matrices are often modified to include the set of observed frequencies in the dataset being examined. Models that take into account the observed amino acid frequencies are often denoted by the '+F' suffix [11]. If we assume that the substitution process is reversible then the number of free parameters is reduced to from 399 to 189. However due the computational burden imposed by optimising all 189 free parameters of the instantaneous rate matrix with large datasets and the risk of overfitting the matrix parameters when analysing small datasets has meant that most phylogeny programs rely on empirical models of protein evolution. Dayhoff et al. [12] were the first to develop a general model of amino acid substitution using the limited amount of sequence data available at the time. Since then, several additional models have been developed from other datasets and using different techniques, such as maximum parsimony and maximum likelihood [13-22].

There has been a great deal of research into various techniques for performing model selection on nucleotide data [23]. In the past, three measures have been used to select the best-fit substitution model. The hierarchical likelihood ratio test (hLRT) consists of a tree hierarchy where the best-fit model is selected by performing a number of pairwise likelihood ratio tests and navigating the tree to arrive at the final model [24]. However the hLRT is only suitable for models which can be defined as subsets of each other, therefore is generally only applied to nucleotide model selection. For example, the F81 model [25] is a subset of the HKY model [26] with the transition and transversion rates set to be equal. As the different amino acid matrices do not have any free parameters, it is not possible to define a similar tree hierarchy as with nucleotide models. The Akaike information criterion (AIC) [27] and Bayesian information criterion (BIC) [28] belong to a different class of model selection measures that compare all of the models simultaneously according to some measure of fitness. Although these measures have been used for many years in nucleotide model selection, only recently have programs such as MODELGENERATOR [29] and Prottest [30] been specifically developed to perform statistical analyses of the complete set of available amino acid substitution models.

Until now many phylogenetic analyses of multiple datasets from a fixed set of taxa have assumed a single substitution model for all sets of homologs (e.g. [31-33]). We argue that if it is assumed that a single amino acid matrix is the best-fit matrix for all genes in a dataset, then there is the possibility that the method may encounter situations like the one mentioned previously and produce suboptimal phylogenies. We have performed experiments using real datasets in order to determine if it is correct to build phylogenies from different genes using the same amino acid matrix. This issue has never been examined before and our results show a large differences in best-fit protein models within all of the datasets analysed. The results presented in this paper raise the question of whether we should be performing full protein model selection analyses prior to any amino acid phylogeny estimation.

## Results

**Figure 1:**
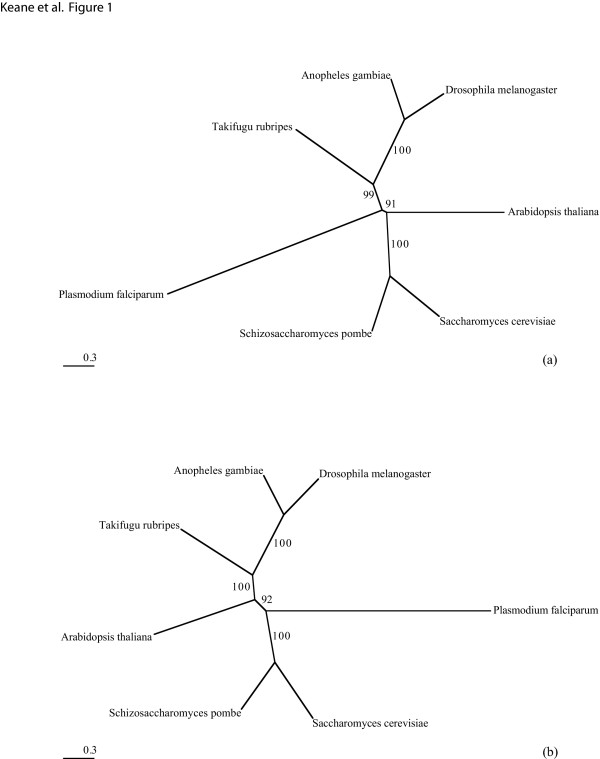
**Alternative Trees**. Two different trees (with bootstrap support values based on 100 replicates) constructed from a single gene family [34] with different protein models using Phyml v2.4.4 [53]. Tree (a) was produced using the MtREV matrix [15] and Tree (b) was produced using the WAG matrix [18].

To investigate the potentially harmful effects of a non-statistical approach to choosing protein models, we built two phylogenies with two arbitrarily selected protein models using a single gene family alignment consisting of 7 taxa (3580 characters in length) taken from the dataset of Philip et al. [34]. Figure 1 shows how it is possible to obtain two different tree topologies both with equally high bootstrap support by arbitrarily choosing the protein substitution model. In situations like this, the high bootstrap support values of both trees might indicate that either one of these alternate trees is optimal. However, bootstrap values can be misleading (as bootstrapping is performed using the same model that was used to construct the phylogeny) and cannot be used to infer information about the suitability of the substitution model [9]. Therefore without detailed prior knowledge of the phylogenetic relationships or first determining the best-fit model from the set of available models, it is difficult to determine the optimal ML tree.

**Figure 2:**
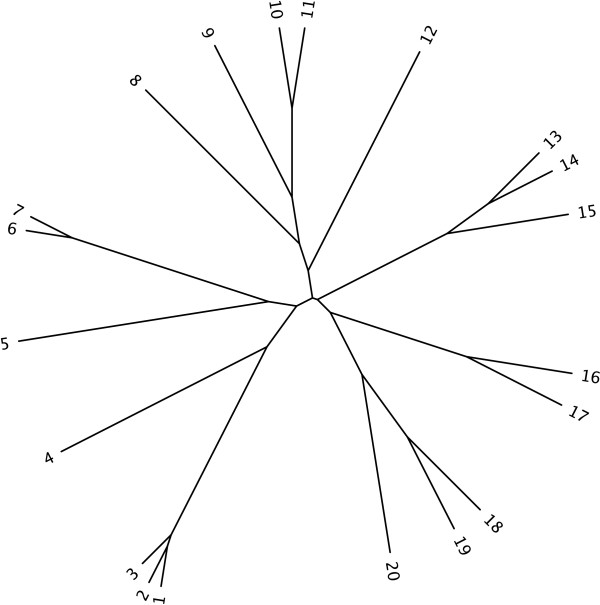
**Base Tree**. The true tree used to generate all of the simulated alignments.

The likelihood is calculated as the probability of obtaining the data (multiple sequence alignment) given the model of evolution (substitution model and phylogeny). Ideally we would prefer to use the true tree when performing our model selection as this would remove any conflicting signals from an incorrect base tree. However on real datasets the true tree is unknown so we must use some approximation of the true tree for the model selection procedure in order to estimate the model parameters [35]. In the following sections, we provide the results of simulations to investigate the effects of using varying base trees, alignments lengths, among-site rate variation (ASRV) parameters, and amino acid frequencies in amino acid alignments on the selection of protein models. For all of the simulations, we used the same 20 taxon clocklike tree (see Figure 2) used by Posada and Crandall [36] to generate our simulated alignments. We also took a number of previously published real datasets for which the model of amino acid substitution is suspected to follow a particular matrix (due to the source of the data) in order to test the accuracy of the selection method. We then examined the extent of amino acid matrix variation among three large sets of orthologs from each of the Domains of Life.

### Base tree sensitivity

The results of the simulations using different base trees (true, random, and NJ-JTT tree) for the model selection procedure are presented in Table 1. The most important observation from the table is that the recovery rates are significantly reduced for almost all models when a random tree is used compared to either the true tree or the NJ tree. However there is one notable exception to this where many of the +I+G alignments display slightly higher recovery rates with a random tree than with either the true tree or NJ tree. Further analysis of our results (not shown) shows that this appears to be due to the fact that when a random tree is used, the model selection procedure tends to generally favour over-parameterised models which is consistent with the findings of a previous study on nucleotide sequences [37]. There is very little difference between using the true tree and an NJ tree which confirms previous findings for nucleotides that a relatively good tree is sufficient for estimating accurate model parameters [35,38,39]. It is also very interesting to note that the correct amino acid matrix was selected in almost every case (data not shown) regardless of the base tree, indicating that the only area of uncertainty in these simulations is the correct choice of ASRV.

**Table 1:** Base Tree Simulations. Results of simulated datasets when a random, NJ-JTT, and the true tree are used as the base tree for the model selection procedure and the sequence length is 500 characters. Each entry is the number of times out of 100 replicates the correct model was selected by each measure.

	Random			NJ-JTT			True		
Model	AIC_1_	AIC_2_	BIC	AIC_1_	AIC_2_	BIC	AIC_1_	AIC_2_	BIC

Blosum	0	0	0	91	98	99	84	96	96
Blosum+I	0	0	0	94	99	100	100	100	100
Blosum+G	58	65	67	75	83	87	75	84	87
Blosum+I+G	89	86	85	90	88	87	89	85	85
CPREV	0	0	0	92	99	100	93	98	99
CPREV+I	0	0	0	98	99	99	97	99	100
CPREV+G	80	83	85	89	89	90	89	90	90
CPREV+I+G	94	91	91	80	75	73	80	75	73
Dayhoff	0	0	0	95	100	100	94	98	99
Dayhoff+I	0	0	0	98	100	100	96	99	100
Dayhoff+G	68	72	74	77	86	90	79	88	91
Dayhoff+I+G	94	93	93	82	74	74	84	74	72
JTT	0	0	0	94	99	100	97	99	99
JTT+I	0	0	0	96	100	100	97	100	100
JTT+G	54	59	62	78	85	86	81	87	89
JTT+I+G	94	94	93	89	85	82	92	87	84
MtREV	0	0	0	85	96	97	94	99	99
MtREV+I	0	0	0	92	99	100	97	100	100
MtREV+G	80	87	87	92	94	95	93	94	94
MtREV+I+G	86	85	84	68	65	61	70	63	63
WAG	0	0	0	88	97	99	95	99	100
WAG+I	0	0	0	98	100	100	96	100	100
WAG+G	74	79	79	83	89	89	83	88	89
WAG+I+G	90	89	87	79	73	69	78	73	71

We next examined the difference in the models selected using the likelihood values from the quick NJ-JTT base tree and those of fully optimised ML phylogenies produced using all of the individual models (see methods). There is very little difference (<10%) between the model selection accuracy when model selection was carried out using a full ML tree search using each available model and the models selected by the quicker NJ-JTT method (see Table 2). For the proteobacteria dataset, the NJ-JTT model selection procedure differed to the full ML analysis selections in fewer than 7% of cases, with most of the different selections being due to selecting the same amino acid rate matrix and different ASRV parameters. The NJ-JTT model selection procedure and ML analysis achieved closer to full agreement in the archaea dataset, where the model predictions given by the two procedures differed in fewer than 5% of cases. There was a similar pattern with the vertebrate dataset where the NJ-JTT model selection procedure differed in fewer than 9% of cases compared to the full ML analysis procedure. Table 2 shows that in the majority of cases where different models were selected, the same amino acid matrix was selected with the difference being due to different selections of optimal ASRV parameters.

**Table 2:** Full ML Comparison. A comparison of the models selected from the likelihood values obtained from a full ML tree search using all models and the likelihood values using the default NJ-JTT base tree. The column 'Identical' indicates the number of times (out of 100 alignments) both procedures selected the same model. The column titled 'Rate' indicates cases when the same amino acid matrix and a different ASRV was selected. The column titled 'Matrix' indicates cases when the a different amino acid matrix was selected.

	AIC_1_			AIC_2_			BIC		
Dataset	Identical	Rate	Matrix	Identical	Rate	Matrix	Identical	Rate	Matrix

Proteobacteria	95	4	1	93	6	1	94	2	4
Archaea	99	1	0	96	2	2	95	2	3
Vertebrate	91	7	2	94	5	1	97	1	2

### Sequence length

One of the factors that is believed to affect the results of the nucleotide model selection is sequence length [36]. We wanted to investigate what effect (if any) sequence length would have on amino acid model selection by performing the model selection procedure on varying length alignments. Table 3 shows the recovery rates of each of the three measures (AIC_1_, AIC_2_, and BIC) for the three different alignment lengths (100, 500, and 1000 characters). As expected, the rates for the longer sequences are increased compared to the shorter sequences. One noticeable feature with the 100 character dataset is that the number of times the correct model was selected when a +I+G ASRV was present was significantly reduced for all matrices. Further examination of the results shows that this is almost always due to the model selection procedure picking the +G version of the model. This is due to the fact that the difference in likelihoods between the +I+G and +G models is quite small at short sequence lengths and not significant enough for the measures to prefer the more parameterised +I+G models. In these cases, we have observed that the *α *parameter of the gamma distribution is generally estimated to be less than 0.2 in order to accommodate the invariable sites. When the sequence length is increased to 1000 characters, the difference between the likelihoods of the +G and +I+G models increases and is enough for the model selection measures to prefer the +I+G models. As the BIC takes into account sample size (sequence length), it is not affected to the same extent by this phenomenon (see Table 3). Again the correct amino acid substitution matrix was selected in almost every case regardless of sequence length (data not shown).

**Table 3:** Alignment Length Simulations. Results of the simulated datasets for alignments of 100, 500, and 1000 characters in length. Each entry is the number of times out of 100 replicates the correct model was selected by each measure (using the default NJ-JTT base tree).

	100			500			1000		
Model	AIC_1_	AIC_2_	BIC	AIC_1_	AIC_2_	BIC	AIC_1_	AIC_2_	BIC

Blosum	86	96	95	91	98	99	94	99	100
Blosum+I	95	100	95	94	99	100	98	100	100
Blosum+G	89	95	95	75	83	87	79	85	88
Blosum+I+G	44	30	30	90	88	87	95	95	94
CPREV	92	99	99	92	99	100	95	100	100
CPREV+I	94	100	100	98	99	99	99	99	100
CPREV+G	87	99	98	89	89	90	91	96	97
CPREV+I+G	51	37	37	80	75	73	95	94	94
Dayhoff	92	99	99	95	100	100	93	99	100
Dayhoff+I	94	100	100	98	100	100	96	100	100
Dayhoff+G	83	93	93	77	86	90	94	94	95
Dayhoff+I+G	54	35	38	82	74	74	95	92	91
JTT	95	98	98	94	99	100	93	98	100
JTT+I	95	99	98	96	100	100	96	100	100
JTT+G	87	94	94	78	85	86	91	91	93
JTT+I+G	48	36	40	89	85	82	96	95	94
MtREV	95	98	98	85	96	97	91	97	97
MtREV+I	97	100	100	92	99	100	97	100	100
MtREV+G	86	97	97	92	94	95	92	95	96
MtREV+I+G	29	17	17	68	65	61	87	85	83
WAG	91	97	96	88	97	99	97	98	100
WAG+I	94	100	99	98	100	100	97	99	100
WAG+G	85	95	93	83	89	89	86	95	95
WAG+I+G	50	34	36	79	73	69	97	96	94

### Among-site rate variation parameters

ASRV parameters can vary greatly in real datasets therefore it is important to investigate if the model selection procedure is affected in any way by varying ASRV's. Table 4 shows the results of the simulations where the gamma shape parameter was varied. The most noticeable trend in the table is the reduction in recovery rates of the +G simulations with higher values of *α*. A closer examination of the results shows that this is due to the model selection measures incorrectly selecting the +I+G ASRV where the true ASRV is +G. It is quite noticeable that the BIC is the least affected measure as it associates a much higher cost for adding more parameters to the model than either of the AIC metrics. Therefore we attribute this reduction in accuracy to be a property of the AIC. This phenomenon is also matched by better results in the +I+G simulations as the values of *α *are increased. This increase in accuracy is consistent with our earlier result (the gamma parameter being significantly reduced in order to incorporate invariable sites at the short sequence length) meaning that at high values of *α*, such as 1.0 or 2.0, the separate invariable sites parameter is explicitly required by the model to account for the proportion of invariable sites. Just like in the previous tables, the correct amino acid matrix was selected in almost every case.

**Table 4:** Gamma Distribution Simulations. Results of simulations when the *α *parameter of the gamma distribution was varied between 0.5, 1.0, and 2.0. The sequence length was kept constant at 500 characters and the proportion of invariable sites was 0.2. Each entry is the number of times out of 100 replicates that the correct model was selected.

	*α *= 0.5			*α *= 1.0			*α *= 2.0		
Model	AIC_1_	AIC_2_	BIC	AIC_1_	AIC_2_	BIC	AIC_1_	AIC_2_	BIC

BLOSUM62+G	75	83	87	32	62	69	36	68	74
BLOSUM62+I+G	90	88	87	95	93	92	100	100	100
CPREV+G	89	89	90	39	72	77	39	65	79
CPREV+I+G	80	75	73	93	89	89	100	100	100
Dayhoff+G	77	86	90	33	36	74	38	60	66
Dayhoff+I+G	82	74	74	98	95	92	100	100	100
JTT+G	78	85	86	43	71	76	25	54	63
JTT+I+G	89	85	82	98	96	94	100	100	100
MtREV+G	92	94	95	46	72	76	51	75	84
MtREV+I+G	68	65	61	90	85	83	100	100	100
WAG+G	83	89	89	35	70	76	32	70	79
WAG+I+G	79	73	69	97	91	90	100	100	100
Dayhoff+I+G	54	35	38	82	74	74	95	92	91
JTT	95	98	98	94	99	100	93	98	100
JTT+I	95	99	98	96	100	100	96	100	100
JTT+G	87	94	94	78	85	86	91	91	93
JTT+I+G	48	36	40	89	85	82	96	95	94
MtREV	95	98	98	85	96	97	91	97	97
MtREV+I	97	100	100	92	99	100	97	100	100
MtREV+G	86	97	97	92	94	95	92	95	96
MtREV+I+G	29	17	17	68	65	61	87	85	83
WAG	91	97	96	88	97	99	97	98	100
WAG+I	94	100	99	98	100	100	97	99	100
WAG+G	85	95	93	83	89	89	86	95	95
WAG+I+G	50	34	36	79	73	69	97	96	94

### Amino acid frequency perturbation

Each of the protein substitution models consists of an instantaneous rate matrix (Q) which includes a set of original amino acid frequencies (*π*_*i*_) obtained from the dataset that was used to generate the model. If we use the observed amino acid frequency parameters of the dataset being examined (denoted by the '+F' suffix) instead, then we include 19 extra free parameters when evaluating each model. We were interested in investigating what effect the change in amino acid frequency proportions would have on the model selection procedure and whether the corresponding '+F' versions of the models would be selected. We would expect our model selection procedure to be robust enough to select the corresponding amino acid matrix despite the variation in amino acid frequencies. Table 5 shows that when the original model amino acid frequencies were randomly perturbed, there was a definite trend among all of the model selection measures to select the corresponding '+F' version of the particular model over the original models. The recovery rates are extremely high across all categories with only a few exceptions. When amino acid frequencies deviate from the default amino acid frequencies of a particular model, there is a trend towards the '+F' version of the same model.

**Table 5:** Amino Acid Frequency Simulations. Results of the simulated datasets where the original amino acid frequencies are randomly perturbed by up to 10% from the original values and the alignment length is 500 characters. Each entry indicates the number of times out of 100 replicates the correct model was selected by each measure.

Model	AIC_1_	AIC_2_	BIC	Model	AIC_1_	AIC_2_	BIC
Blosum+F	94	100	100	JTT+F	93	100	100
Blosum+I+F	71	91	95	JTT+I+F	67	89	94
Blosum+G+F	86	93	96	JTT+G+F	75	89	92
Blosum+I+G+F	99	97	96	JTT+I+G+F	98	96	96
CPREV+F	92	100	100	MtREV+F	93	99	99
CPREV+I+F	87	98	99	MtREV+I+F	86	96	99
CPREV+G+F	93	96	97	MtREV+G+F	86	93	95
CPREV+I+G+F	89	87	84	MtREV+I+G+F	85	82	80
Dayhoff+F	93	99	99	WAG+F	95	100	100
Dayhoff+I+F	91	98	99	WAG+I+F	82	96	97
Dayhoff+G+F	86	93	96	WAG+G+F	88	95	96
Dayhoff+I+G+F	99	97	96	WAG+I+G+F	90	89	89

### Expected model selections

Some of the amino acid substitution matrices were developed specifically for use with certain types of datasets. For example, the MtREV [15] and MtMam [16] models were developed from different mitochondrial datasets. The RtREV matrix was developed specifically for use with retroviral and reverse transcriptase datasets [19]. Indeed the RtREV authors presented a study showing how the RtREV matrix consistently produced higher likelihoods than other matrices such as JTT and WAG for specific datasets. Consequently it is expected that these matrices will perform quite well during model selection when applied to datasets of similar origin to the original datasets used to develop these models. The results of the model selection procedure for a number of datasets where the substitution process is known are outlined in Table 6. We have provided a column that describes the expected amino acid matrix (based on previously published information on each of the alignments). It is clear that there is a noticeable bias in each of the datasets towards some form of the expected amino acid matrix.

**Table 6:** Real Dataset Analysis. Results of the model selection on the specialised datasets (see the references for full descriptions of the individual datasets). Amino acid matrix expectations are based on previously published information about the sequences ([19, 54, 55] and LANL [56]).

Dataset	Source	Expected	AIC_1_	AIC_2_	BIC
mtCDNApri	Yang [54]	MtMam	MtMam+I+G	MtMam+G	MtMam+G
mtCDNAape	Yang [54]	MtMam	MtMam+F	MtMam+F	MtMam+F
70pep_nogap	Reyes *et al*. [55]	MtMam	MtMam+I+G+F	MtMam+I+G	MtMam+I+G
BETA	Dimmic *et al*. [19]	RtREV	RtREV+G+F	RtREV+G	RtREV+G
ENDO	Dimmic *et al*. [19]	RtREV	RtREV+I+G+F	RtREV+I+G+F	RtREV+I+G+F
GAGGAM	Dimmic *et al*. [19]	JTT	JTT+G+F	JTT+G+F	JTT+G+F
GAGHIV	Dimmic *et al*. [19]	JTT	JTT+G+F	JTT+G+F	JTT+G+F
GAMMA	Dimmic *et al*. [19]	RtREV	CPREV+G+F	RtREV+G	RtREV+G
LENTI	Dimmic *et al*. [19]	RtREV	RtREV+I+G+F	RtREV+I+G+F	RtREV+I+G+F
SPUMA	Dimmic *et al*. [19]	RtREV	RtREV+G	RtREV+G	RtREV+G
NONLTR	Dimmic *et al*. [19]	RtREV	RtREV+I+G+F	RtREV+I+G+F	RtREV+I+G+F
SIVPOLPRO	LANL	RtREV	RtREV+G+F	RtREV+G+F	RtREV+G

### Model variation among multi-gene datasets

**Figure 3:**
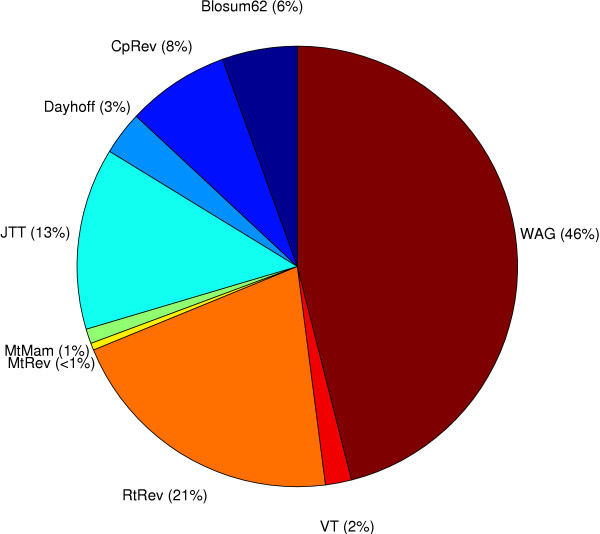
**Proteobacteria Dataset**. A break-down of the set of best-fit protein models for the proteobacteria dataset.

**Figure 4:**
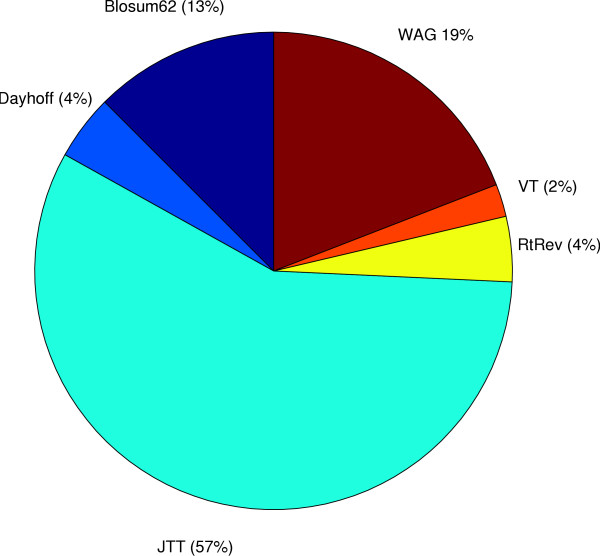
**Vertebrate Dataset**. A break-down of the set of best-fit protein models for the vertebrate dataset.

**Figure 5:**
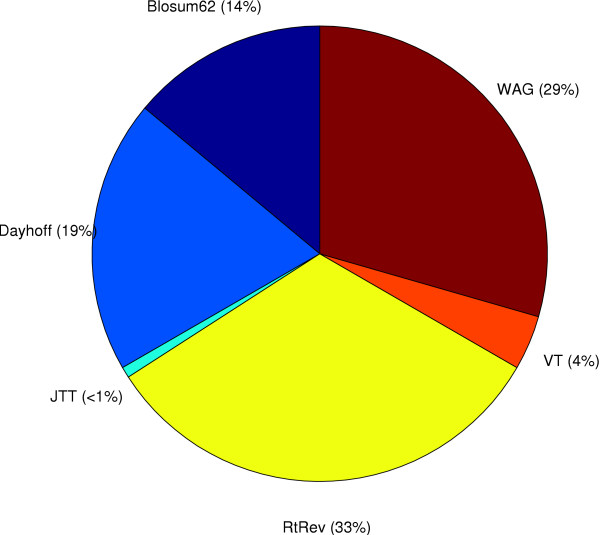
**Archaea Dataset**. A break-down of the set of best-fit protein models for the archaea dataset.

Figure 3 shows that for the 2135 proteobacteria orthologs the WAG matrix was selected for approximately 46% of the genes, the RtREV matrix was optimal for the second largest proportion (21%), and a large number of other models best described the other 33% of the genes. The vertebrate dataset (Figure 4) displays a different pattern to the bacterial dataset with the JTT matrix making up 57% of the best-fit models and the WAG matrix making up a much smaller proportion (19%) of models. The most dominant substitution matrix in the archaea dataset (Figure 5) is the RtREV matrix (33%) with the WAG (29%) matrix close behind with the rest of the genes fitting a selection of other models. It is interesting to note the default scoring model used by ClustalW (Blosum62) to align the sequences did not feature very often in the set of optimal matrices. This suggests that the scoring model used for the alignment procedure does not bias the selection of the optimal substitution matrix. A global examination of the figures shows that no single model emerges from the rankings as the overall best overall model for any of the datasets.

### Model selection and tree accuracy

Table 7 shows that when we generated simulated alignments with one particular model and then built phylogenies using each of the other available models, the Robinson-Foulds (RF) distances [40] were either equal or worse than when we built phylogenies using the same model that was used to generate the alignments. These results show that in simulations the choice of protein model has a definite effect the topology of the inferred tree.

**Table 7:** Tree Accuracy Simulations. Results of the simulated tree accuracy test where alignments were generated with a particular model and then phylogenies were built using all of the other available models. Each entry is the average scaled Robinson-Foulds (RF) distance [40] over the trees inferred using the alternative models. This test was repeated 10 times for each model and the values in brackets are the RF distances from the true tree when phylogenies were inferred using the model that generated the alignment. Phyml [53] was used to build all trees.

Model	RF Distance	Model	RF Distance
Blosum	0.03 (0.03)	JTT	0.05 (0.05)
Blosum+I	0.02 (0.02)	JTT+I	0.05 (0.04)
Blosum+G	0.08 (0.06)	JTT+G	0.04 (0.03)
Blosum+I+G	0.05 (0.05)	JTT+I+G	0.12 (0.11)
CPREV	0.05 (0.04)	MtREV	0.06 (0.05)
CPREV+I	0.09 (0.04)	MtREV+I	0.08 (0.08)
CPREV+G	0.06 (0.05)	MtREV+G	0.07 (0.06)
CPREV+I+G	0.07 (0.06)	MtREV+I+G	0.12 (0.1)
Dayhoff	0.07 (0.07)	WAG	0.02 (0.02)
Dayhoff+I	0.06 (0.05)	WAG+I	0.04 (0.04)
Dayhoff+G	0.06 (0.06)	WAG+G	0.1 (0.1)
Dayhoff+I+G	0.05 (0.04)	WAG+I+G	0.04 (0.04)

In real datasets, the true tree is unknown and therefore it is impossible to know with certainty if we have found the true tree. One possible indication as to whether the choice of model is improving the inferred phylogenies might be to take a large dataset of orthologs and measure the level of congruence among the inferred trees. It would be expected that the congruence among the trees would increase as the optimal models are used to build the trees. We took our proteobacteria dataset (2135 orthologs) and built phylogenies using fixed amino acid matrices and also built phylogenies using the optimal protein model for each alignment. Table 8 shows that for the proteobacteria dataset when the optimal models were used to infer the trees, the median RF distance was lower than using a fixed model in the majority of cases.

**Table 8:** Proteobacteria Tree Accuracy Analysis. The scaled Robinson-Foulds (RF) distances [40] of the trees produced from the Proteobacteria dataset using fixing a model used to build trees from each alignment. The values reported are the median and average distance computed by comparing every tree against every other tree. When the optimal set of models were used the median was 0.22 and the average was 0.34. Phyml [53] was used to build all trees.

Model	Median RF	Mean RF	Model	Median RF	Mean RF
Blosum	0.23	0.35	JTT	0.23	0.34
Blosum+I	0.25	0.35	JTT+I	0.25	0.35
Blosum+G	0.25	0.35	JTT+G	0.25	0.35
Blosum+I+G	0.25	0.35	JTT+I+G	0.25	0.35
CPREV	0.24	0.35	MtREV	0.25	0.35
CPREV+I	0.25	0.35	MtREV+I	0.25	0.35
CPREV+G	0.25	0.35	MtREV+G	0.25	0.35
CPREV+I+G	0.25	0.35	MtREV+I+G	0.25	0.35
Dayhoff	0.2	0.34	WAG	0.21	0.34
Dayhoff+I	0.21	0.34	WAG+I	0.23	0.35
Dayhoff+G	0.22	0.34	WAG+G	0.25	0.35
Dayhoff+I+G	0.22	0.34	WAG+I+G	0.25	0.35

## Discussion

We have studied the influence of various factors on protein model selection. Our simulations have confirmed previous work showing that the model selection procedure performs quite accurately using an approximate tree for model selection. One of the most interesting results that we have shown using real datasets is that less than 9% of the time was a different matrix selected using a full ML analysis than those selected using a quick NJ-JTT method. This further strengthens the recent results presented by Sullivan *et al*. [38] showing that successive-approximation methodologies to phylogeny estimation does not suffer from any significant loss in accuracy. Our simulations have also shown that protein model selection is not as sensitive as nucleotide model selection to sequence length differences. Recovery rates remain relatively constant over different sequence lengths with the only exception to this being at short sequence lengths when the difference in likelihood values can result in an overly-simplistic model being selected (+G instead of +I+G). We have also shown that when amino acid frequencies deviate from the default amino acid frequencies of each model, there is a clear trend towards the '+F' version of each model. This phenomenon was also observed in the results of the real dataset analysis presented in Table 5 with many of the real datasets being best described by '+F' versions of the expected models. One constant trend across all of the simulations we have performed is that the correct amino acid matrix is selected by both measures close to 100% of the time regardless of factors such as base tree accuracy, sequence length, ASRV variances, or amino acid frequencies.

It should be emphasized that many of the current set of models of amino acid or nucleotide substitution make many unrealistic assumptions such as reversibility, amino acid composition stationarity, and homogeneous substitution rates. However much work is currently taking place to develop methods to loosen many of these restrictions [41-43]. While the focus of this work has been to demonstrate the usefulness of performing protein model selection, it must be stated that model selection measures can only provide information on which of the given set of models best-fits the data and cannot give any indication of how close a particular model is to reality.

We have highlighted an example where two highly-supported and topologically different phylogenies were produced from the same alignment using two arbitrarily selected amino acid substitution matrices (see Fig. 1). The likelihood values of the two trees are -30722 for the MtREV tree and -28996 for the WAG tree with the extremely high bootstrap support values providing evidence that the observed trees are not due to a stochastic error (e.g. the treesearch getting stuck in a local optima). To further rule out any source of stochastic error, the corresponding likelihoods for the MtREV tree with the WAG matrix is -29288 and -30959 for the WAG tree with the MtREV matrix, thereby confirming that both matrices favor different tree topologies. A tree constructed using the optimal model for this alignment (RtREV+I+G+F) agrees with the WAG tree. At first glance, our particular example may seem slightly unrealistic as we have used a mitochondrial model to construct a tree from nuclear genes. However, as we have shown, one of the best models for proteobacterial and archaeal genes is frequently (22% and 33% of the time respectively) a model that was derived from retroviral Pol proteins. Therefore, *ad hoc *model selection, even when using arguments about the origin of the model (nuclear versus organelle, or some such) are still *ad hoc *arguments. The maximum likelihood principle suggests the use of the best of the available models and in some cases, the best performing model can be surprising.

The results of our cross-domain substitution model analysis are interesting as there are noticeable differences in the groups of models selected by each dataset with no single matrix emerging as the best for any of the datasets. The large diversity of amino acid matrices cannot come as a great surprise as it would seem intuitively unreasonable to assume that a very large group of independently evolving gene families from a fixed taxon set followed an identical amino acid substitution pattern. Perhaps one of the most significant findings is that the RtREV matrix [19] features so prominently in both the proteobacteria and archaea datasets. The WAG matrix [18], for instance was derived from a globular protein dataset and was shown to produce higher likelihood values in general, compared with the JTT matrix for the dataset from which it was derived. This seemed to indicate that choosing a matrix based upon the method or the data used to derive the matrix might be a good idea. However, our finding that for so many alignments from cellular life, the best matrix was derived from viral sequences is surprising and the consequence is that *ad hoc *arguments for choice of matrix may not reasonable.

## Conclusion

In this study, we have analysed the ability of the AIC and the BIC to select the appropriate evolutionary model in cases where the model is known. We have shown that both methods are suitable for this purpose. We have also shown that none of the currently available models is universally preferred for all alignments and that there is considerable variation in the substitution process across protein families. What we have not attempted to show is that for any given alignment the selected model is the actual model that gave rise to the observed data. However, on the basis of our results we can speculate on the appropriateness of the models. Considering that a viral model is one of the most preferred models for these cellular sequences, perhaps none of the models are really capturing the data. The models are homogeneous across the tree and this is likely to be a simplification. Therefore, even though we have produced a robust method of model selection, it is likely that the models themselves need to be improved.

## Methods

The AIC is a popular model selection measure that attempts to strike a balance between the goodness-of-fit and complexity of a model. The AIC is calculated by

*AIC*_1 _= -2 ln *L*_*i *_+ 2*N*_*i*_,     (1)

where *N*_*i *_is the number of free parameters in model *i *and *L*_*i *_is the likelihood value of model *i*. Posada and Crandall [36] presented evidence to show that the more empirically tuned AIC_2 _can sometimes be more accurate at determining the correct nucleotide substitution model. It is calculated by replacing the 2N_*i *_term with 5N_*i *_thus further penalising models of greater complexity. The BIC is another model selection measure and is equivalent to selecting the model with the maximum posterior probability and is calculated from

*BIC *= -2 ln *L*_*i *_+ *N*_*i *_ln *n*,     (2)

where *n *is the sample size (sequence length). The AIC and BIC select the best model by choosing the model with the minimum criterion value. The main difference between the three measures is that the AIC_2 _and BIC tend to select simpler models than the AIC_1 _because they penalise the addition of further model parameters more than the AIC_1 _[44]. If the models that rank highest for a given dataset all include a certain ASRV parameter, then the AIC and BIC will essentially become an ordering with respect to the likelihood values.

We have recently developed a protein model selection program called MODELGENERATOR [29]. It initially constructs a neighbor-joining (NJ) tree using an arbitrary model (default is Jukes-Cantor [45] for nucleotides and JTT [14] for proteins) in order to get an initial base tree for comparison of models. For each model examined, the branch lengths of the tree and model parameters are optimised independently using the PAL library [46]. The program supports 10 amino acid matrices and 14 nucleotide models with either a proportion of invariable sites (+I), gamma shape ASRV (+G), combined invariable and gamma distribution (+I+G), and for amino acids the observed amino acid frequencies (+F). When all matrix and ASRV permutations are considered, a total of 56 nucleotide and 80 protein models can be derived. In the following subsections, we outline how we investigated the effects of various properties of amino acid alignments on the three non-nested model selection measures (AIC_1_, AIC_2_, BIC) when applied to protein model selection. For all of the simulations, we used the same 20 taxon clocklike tree used by Posada and Crandall [36] and the program Seq-Gen vl.3.2 [47] to generate all simulated protein alignments. The presence or absence of a molecular clock in the base tree has been shown to have a negligible effect on the model selection procedure [36]. For all of the simulated and real data tests performed below, MODELGENERATOR was not constrained *a priori *and the full set of 80 protein models was examined during every execution.

### Base tree sensitivity

In order to compare the sensitivity of protein model selection to the accuracy of the base tree, we generated 2400 individual alignments of 500 characters in length using each of the protein models available in Seq-Gen (100 alignments per model) fixing the proportion of invariable sites at 0.2 and the *α *parameter of the gamma distribution to 0.5 where appropriate and then performed model selection using three different base trees – the true tree, an NJ-JTT tree, and a randomly generated tree.

To further investigate the effect of using a distance-based tree for comparison rather than the fully optimised ML tree of each model, we obtained three real datasets from each of the Domains of life. The first dataset consists of 2135 gene families obtained from 25 complete proteobacteria genomes. The homologs were identified by performing all-against-all blast searches [48] of the 25 fully completed genomes with an e-value cutoff of 10^-7^. The sequences were aligned using ClustalW 1.81 [49] with the parameters unchanged from their default settings. The alignments were manually edited to remove badly aligned areas and large gapped areas. The second dataset consisted of amino acid sequences of 16 archaeal genomes retrieved from the COGENT database [50] and one (*Haloarcula marismortui*) from the NCBI. We identified gene families where all members of the family were capable of identifying all other members of the family during database searches (with an e-value cutoff of 10^-7^). Each of these families consisted of between 4 and 16 taxa and were aligned using ClustalW 1.81 using the default settings [49]. The final dataset is a previously published set of 118 vertebrate gene families which included representatives of all the major vertebrate groups obtained from the HOVERGEN database [51] with each alignment consisting of between 4 and 58 taxa [52]. For each dataset, we took a subset of 100 alignments and used Phyml [53] to construct fully optimised ML phylogenies with each of the available protein models and recorded the final likelihood of each individual phylogeny. We limited the ML analysis to 100 randomly-chosen alignments from each dataset due to excessive execution times for the full ML analyses. We took the final likelihood values produced by Phyml and determined the best-fit model and then compared the selected models with those produced by the NJ-JTT model selection procedure.

### Sequence length

We generated 100 replicate alignments of each of the protein models available in Seq-Gen consisting of 100, 500, and 1000 characters in length. For these tests, we fixed the proportion of invariable sites at 0.2 and the *α *parameter of the gamma distribution to 0.5 where appropriate. We performed model estimation using MODELGENERATOR and recorded the model selected by each of the available tests (AIC1, AIC2, BIC).

### Rate-distribution parameters

In order to investigate the possible effect of varying ASRV parameters, we generated a number of different simulated datasets (100 replicate alignments per model) with a fixed sequence length of 500 characters and varied the *α *parameter of the gamma ASRV between 0.5, 1.0, and 2.0 (corresponding to high, medium, and low rate heterogeneity).

### Amino acid frequency perturbation

**Figure 6:**
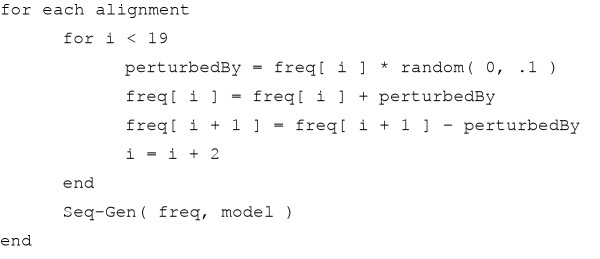
**Pseudo Code**. The algorithm used to generate the simulated +F alignments can be described in pseudocode as follows. The function random returns a random number greater than the first argument and less than the second argument.

In order to create these simulated '+F' alignments, we took the original amino acid frequencies of each model and randomly perturbed each of the individual amino acid frequencies by up to 10% change from its original value in each model (ensuring that the summation of the new set of frequencies remained 1.0) and then used Seq-Gen to generate an alignment using the new set of amino acid frequencies according to the substitution process of the individual model (see algorithm in Figure 6). We generated a total of 2400 alignments of each of the protein models available in Seq-Gen (100 alignments per model) consisting of 500 characters in length in this way so that each individual alignment has a unique set of amino acid frequencies. This analysis more accurately simulates real datasets where the amino acid frequency proportions may differ significantly from the corresponding best-fit model's amino acid frequencies.

### Expected model selection

We obtained the two primate mitochondrial datasets that are included as example datasets in Paml 3.14 [54], namely the files mtCDNApri.aa and mtCDNAape.nuc (translated the nucleotide sequences to amino acids). We also obtained the amino acid sequences of a recent study examining congruence among mammalian mitochondrial and nuclear genes [55]. We downloaded a copy of the complete test dataset used in the creation and testing of the RtREV matrix [19]. We also obtained a Pol alignment from the 2003 HIV and SIV alignments database [56] and performed model selection on all of the sequences mentioned.

### Model variation among empirical datasets

For this test, we used the full set of sequences from the three real datasets of the each Domain of life (as described above). We performed model prediction for each alignment in the datasets in order to assess the extent of model differences within the gene families.

### Model selection and tree accuracy

To test for the effect of *ad hoc *model selection on tree accuracy, we performed an analysis on both simulated and real data. In the simulated analysis, we generated alignments of 500 characters in length using all of the amino acid matrices and rate distributions setting the proportion of invariable sites to 0.3 and the *α *parameter of the gamma distribution to 0.5 where appropriate. The base tree in Figure 2 was used to generate all alignments. We then analysed each alignment using all of the possible models except the one used to generate the alignment. We repeated this test 10 times for each model and computed the average scaled RF distance [40] from all the inferred trees to the true tree for each model. We then build phylogenies using the same model as was used to generate each alignment and reported the RF distance to the true tree also.

In an attempt to analyse the effect of *ad hoc *model selection on tree accuracy on real data, we took the proteobacteria dataset (2135 orthologs) and built phylogenies by using each of the available models as a fixed model for the entire dataset. We recorded the median and average of the all-against-all RF distances of the trees using Clann v3.0.3 [57]. For all possible pairs of trees, Clann prunes the taxa of the trees so that only the common taxa are left and then computes the scaled RF distance.

### Supplementary data

All of the simulated and real alignments mentioned in the paper are available for download from [58].

## Authors' contributions

TMK and JOM initially formulated the idea for the manuscript. CJC and MMP provide some of the real datsets used in the analyses. TMK developed the software, performed the experiments, and drafted the manuscript. All authors read and approved the final manuscript.
